# Author Correction: Disrupting cellular memory to overcome drug resistance

**DOI:** 10.1038/s41467-024-48797-x

**Published:** 2024-05-20

**Authors:** Guillaume Harmange, Raúl A. Reyes Hueros, Dylan L. Schaff, Benjamin Emert, Michael Saint-Antoine, Laura C. Kim, Zijian Niu, Shivani Nellore, Mitchell E. Fane, Gretchen M. Alicea, Ashani T. Weeraratna, M. Celeste Simon, Abhyudai Singh, Sydney M. Shaffer

**Affiliations:** 1 Cellular and Molecular Biology Graduate Group, Perelman School of Medicine, University of Pennsylvania, Philadelphia, PA USA; 2 Department of Biochemistry and Molecular Biophysics, Perelman School of Medicine, University of Pennsylvania, Philadelphia, PA USA; 3 Department of Bioengineering, School of Engineering and Applied Sciences, University of Pennsylvania, Philadelphia, PA USA; 4 Division of Biology and Biological Engineering, California Institute of Technology, Pasadena, CA 91125 USA; 5 Department of Electrical and Computer Engineering, University of Delaware, Newark, DE 19716 USA; 6 Abramson Family Cancer Research Institute, Perelman School of Medicine, University of Pennsylvania, Philadelphia, PA USA; 7 Department of Chemistry, College of the Arts and Sciences, University of Pennsylvania, Philadelphia, PA USA; 8 Department of Physics, College of the Arts and Sciences, University of Pennsylvania, Philadelphia, PA USA; 9 Department of Biology, College of the Arts and Sciences, University of Pennsylvania, Philadelphia, PA USA; 10 The Wharton School, University of Pennsylvania, Philadelphia, PA USA; 11 Cancer Signaling and Microenvironment Research Program, Fox Chase Cancer Center, Philadelphia, PA USA; 12 Department of Biochemistry and Molecular Biology, Johns Hopkins School of Public Health, Baltimore, MD USA; 13 Sidney Kimmel Cancer Center, Johns Hopkins School of Medicine, Baltimore, MD USA; 14 Department of Cell and Developmental Biology, Perelman School of Medicine, University of Pennsylvania, Philadelphia, PA USA; 15 Department of Pathology, Perelman School of Medicine, University of Pennsylvania, Philadelphia, PA USA

**Keywords:** Systems analysis, Cancer therapeutic resistance, Gene expression, Melanoma, Population dynamics

Correction to: *Nature Communications* 10.1038/s41467-023-41811-8, published online 06 November 2023

The original version of the Supplementary Information associated with this Article contained an error in Supplementary Fig. 9 in which the incorrect full western blots were shown for Supplementary Fig. 4E. The correct blots are now updated in Supplementary Fig. 9, and the replicate blots. The HTML has been updated to include a corrected version of the Supplementary Information.

### Supplementary information

Supplementary Information

