# Supplementary material for: Disrupting cellular memory to overcome drug resistance
Source: Nat Commun. 2023 Nov 6;14:7130. doi: 10.1038/s41467-023-41811-8 (PMC10628298; doi:10.1038/s41467-023-41811-8)
Supplement: Supplementary file 1 — Supplementary Information [file 41467_2023_41811_MOESM1_ESM.pdf]

Supplementary Figure 1

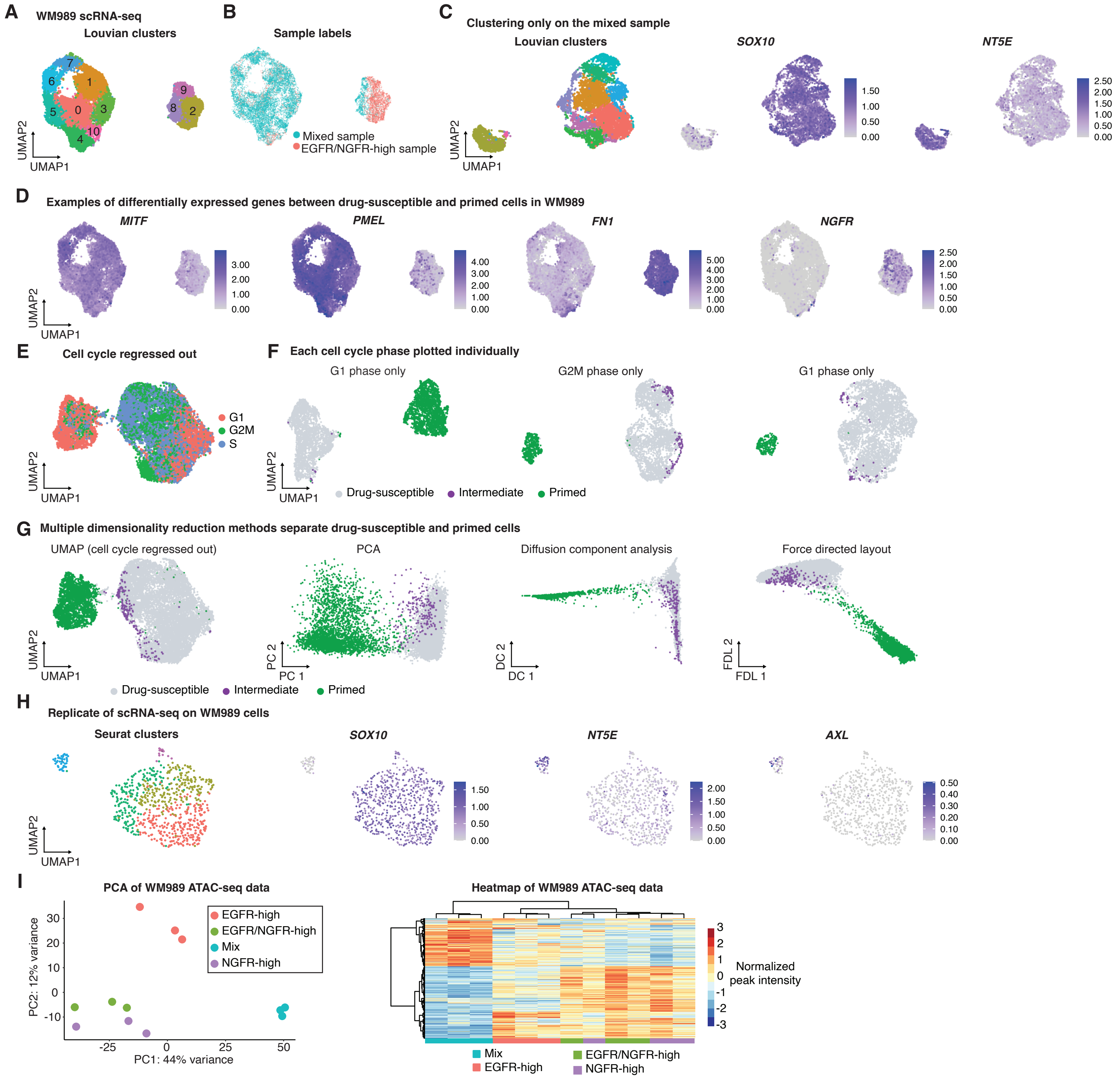

Supplementary Figure 1: Additional characterization of primed cells by scRNA-seq and ATAC-seq. A. Louvain clusters of drug-naive WM989 cells displayed in UMAP space. Clusters 2, 8, and 9 are labeled as primed cells based on their expression of known primed marker genes and *NT5E*. B. UMAP plot showing sample labels from the experiment in Fig. 1C. Each cell is labeled either from the mixed sample or from the sample that was enriched for EGFR/NGFR-high cells. The sample that was enriched for EGFR/NGFR-high cells is significantly enriched in the right cluster containing the primed cells. C. UMAP plots showing only the cells from the mixed sample (does not include the sample enriched for primed cells). Without including the sample that is enriched for primed cells, the drug-susceptible and primed cells (identified by *SOX10* and *NT5E* respectively) still separate into distinct clusters. D. UMAP plots showing log10 normalized gene expression of the indicated gene for each cell. We find that melanocyte markers *MITF* and *PMEL* are high in the drug-susceptible cells, while *NGFR* and *FN1*, markers associated with drug resistance, are high in primed cells. Notably, NGFR-high cells appear in a different location of the right-hand cluster than *AXL* and *EGFR* in Fig. 1D. E. UMAP plot of the same cells shown in Supp. Fig. 1A but with the cell cycle regressed out. The cells are labeled by their phase in the cell cycle. This plot shows that the cell cycle is not driving the separation of the primed and drug-susceptible cells. F. UMAP plots showing cells from each phase of the cell cycle plotted independently. Each phase of the cell cycle separates the drug-susceptible and primed cells, indicating that the separation of these states is not driven by the cell cycle. G. Plots of scRNA-seq data using different dimensionality reduction methods, with cells color-coded by their state. Each of these methods effectively separates drug-susceptible and primed cells, although identifying cells in intermediate states remains challenging across all plots. H. We replicated the scRNA-seq on WM989 cells enriching for primed cells using NT5E as a marker. UMAP plots showing Louvain clustering and gene expression for this replicate. As in the previous scRNA-seq of WM989, we can clearly distinguish the drug-susceptible and primed cells and they show differential expression of the same genes. I. We performed ATAC-seq on a population of sorted mixed, EGFR-high, NGFR-high, and EGFR/NGFR-high WM989 cells. Each condition had three replicates. The scatter plot shows PCA analysis of differential peaks across the samples. The heatmap shows the top 2,000 differential peaks across samples. The order of both rows and columns of the heatmap was determined by Ward's minimum variance clustering. These plots show that although the major difference is between mixed and primed cells, there are consistent differences between EGFR and NGFR-high cells.

Supplementary Figure 2

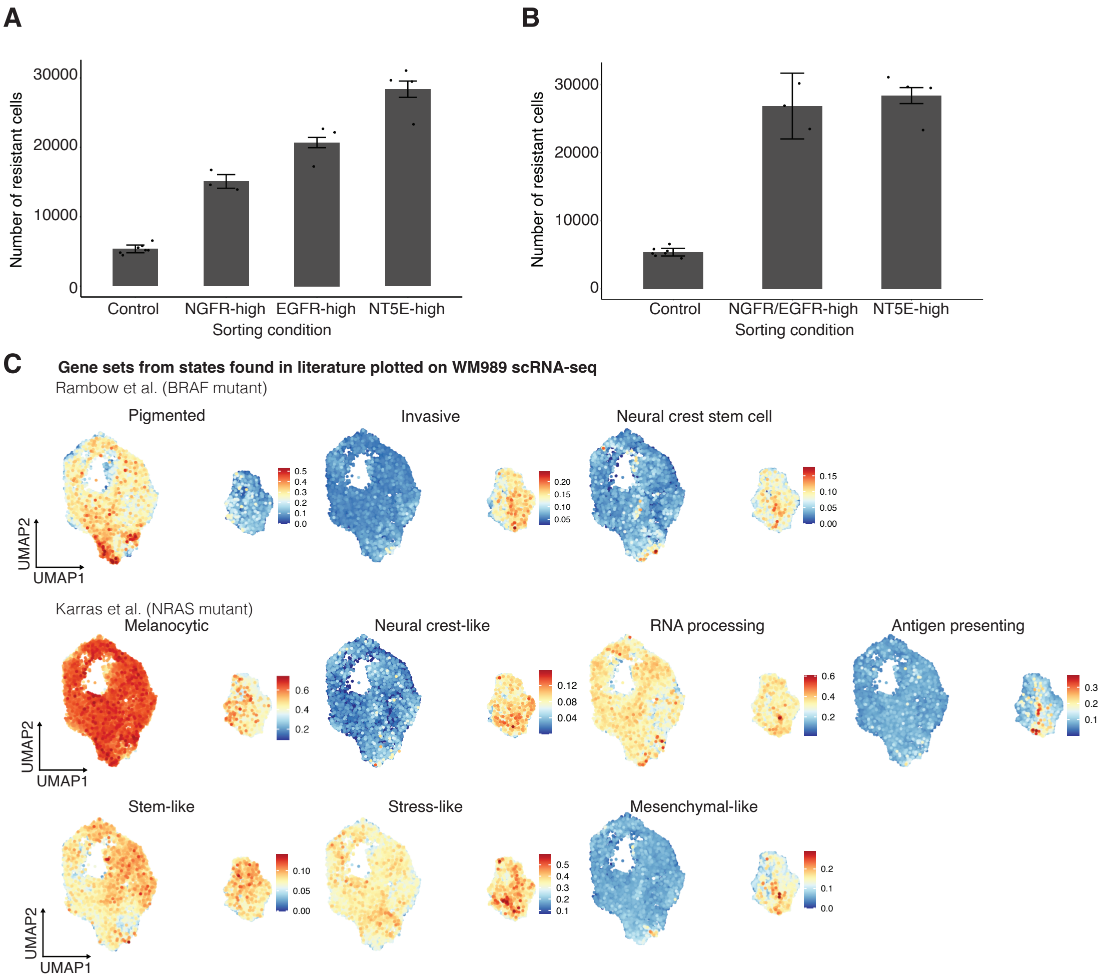

Supplementary Figure 2: Validation of NT5E as a primed state marker and comparison of the primed state to previously defined states. A. We sorted cells based on NGFR (top .2%), EGFR (top .2%), or NT5E (top 2%) expression and then treated them with BRAFi. Bar graph shows how many cells survived after 3 weeks of BRAFi. Control is the bottom 50% of NGFR, EGFR, or NT5E-expressing cells. Each data point is from a separate well representing technical replicates. Error bars are the mean absolute deviation, n=2 biological replicates. B. We sorted cells based on NGFR and EGFR together (top .2%) or NT5E (top 2%) expression and then treated them with BRAFi. Bar graphs show the number of cells after 3 weeks of BRAFi. Control is the bottom 50% of NGFR and EGFR together or NT5E-expressing cells. Each data point is from a separate well representing technical replicates. Error bars are the mean absolute deviation, n=2 biological replicates. C. UMAP plots showing gene set signature scores for melanoma gene expression states previously identified in the literature[1,2]. We see that primed cells express a mix of gene sets previously associated with drug resistance. For the box plots in A and B, the center line is the median, the box is the IQR, and the whiskers indicate 1.5 times the upper and lower IQR. All data points are shown.

Supplementary Figure 3

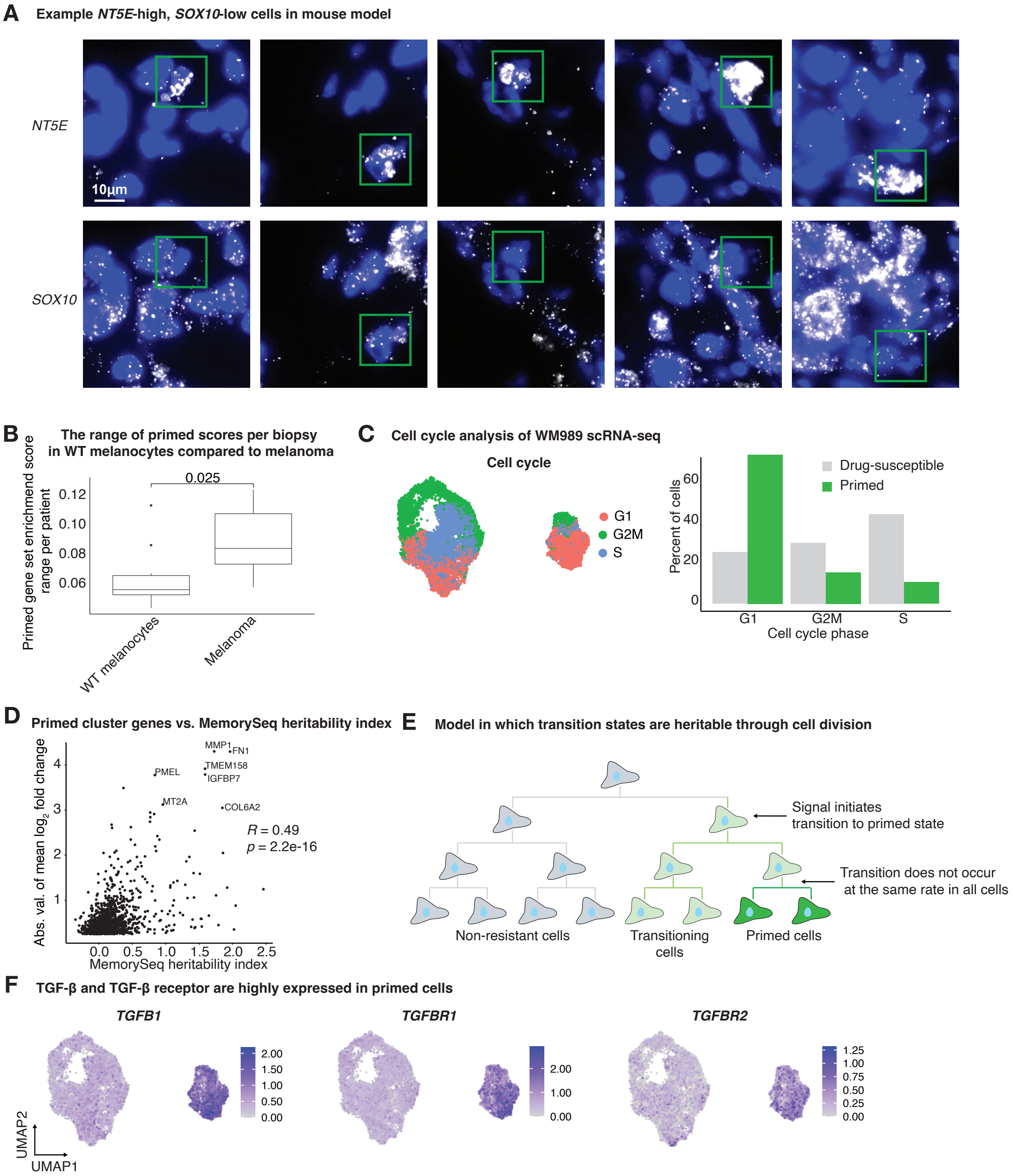

Supplementary Figure 3: The primed state exists in vivo and has memory. A. Additional images of HCR RNA FISH of *NT5E* and *SOX10* in a mouse PDX drug-naive tumor shown in Fig. 3A. Images show rare cells with high levels of *NT5E* and low *SOX10*, indicating that primed cells exist in vivo. The green boxes highlight specific cells in the experiment. DAPI is shown in blue and the RNA FISH HCR is displayed in white. Images acquired at 60X magnification. The scale bar represents 10µm. Assay performed on three slides from one PDX tumor. B. Box plot showing the ranges of primed gene set enrichment scores (as determined by Ucell) per biopsy in patient melanoma cells from Fig. 2D and E compared to those of healthy human melanocytes[3,4,5]. This data set includes 7 melanoma and 14 wild-type melanocyte biopsies. In this plot, the center line is the median, the box is the IQR, and the whiskers indicate 1.5 times the upper and lower IQR. All data points are shown. C. UMAP plot showing the estimated cell cycle stage of each cell. Bar graph showing the quantification of this data highlighting the comparison in cell cycle between the cells in the drug-susceptible and primed clusters. The high proportion of primed cells in G1 suggests they are cycling slower than drug-susceptible cells. D. Scatter plot of the quantification of memory for each gene based on previously published bulk MemorySeq[6] (x-axis) and the differential expression between primed and drug-susceptible cells in the scRNA-seq (y-axis). From the MemorySeq data, we plotted the residual of the fit between the coefficient of variation and mean expression for each gene (described in the paper as the heritability index in which the higher the residual, the more is memory attributed to that gene), and on the y-axis, we plotted the fold change expression between the primed and drug-susceptible states. These two metrics correlate well showing that scRNA-seq comparison between the primed and drug-susceptible clusters identifies the same memory states. E. Schematic of a lineage tree showing how state switching can occur within a lineage from the drug-susceptible to the primed state. Notably, the transition from the drug-susceptible state to the primed state does not occur at the exact same rate in all the branches of the tree. The different rates of progression is important because it allows us to take a measurement at a single point in time at the end of the tree and capture cells undergoing the transition to the primed state. This, along with the assumption that the intermediate state has memory, is what makes it possible to capture the gene expression of transitioning cells in lineages that are crossing. F. UMAP plots showing normalized gene expression of *TGFB1*, *TGFBR1*, and *TGFBR2*. These three genes are all up-regulated in primed cells, suggesting there is likely increased TGF-β signaling in primed cells through autocrine signaling, and potentially some paracrine signaling as well.

Supplementary Figure 4

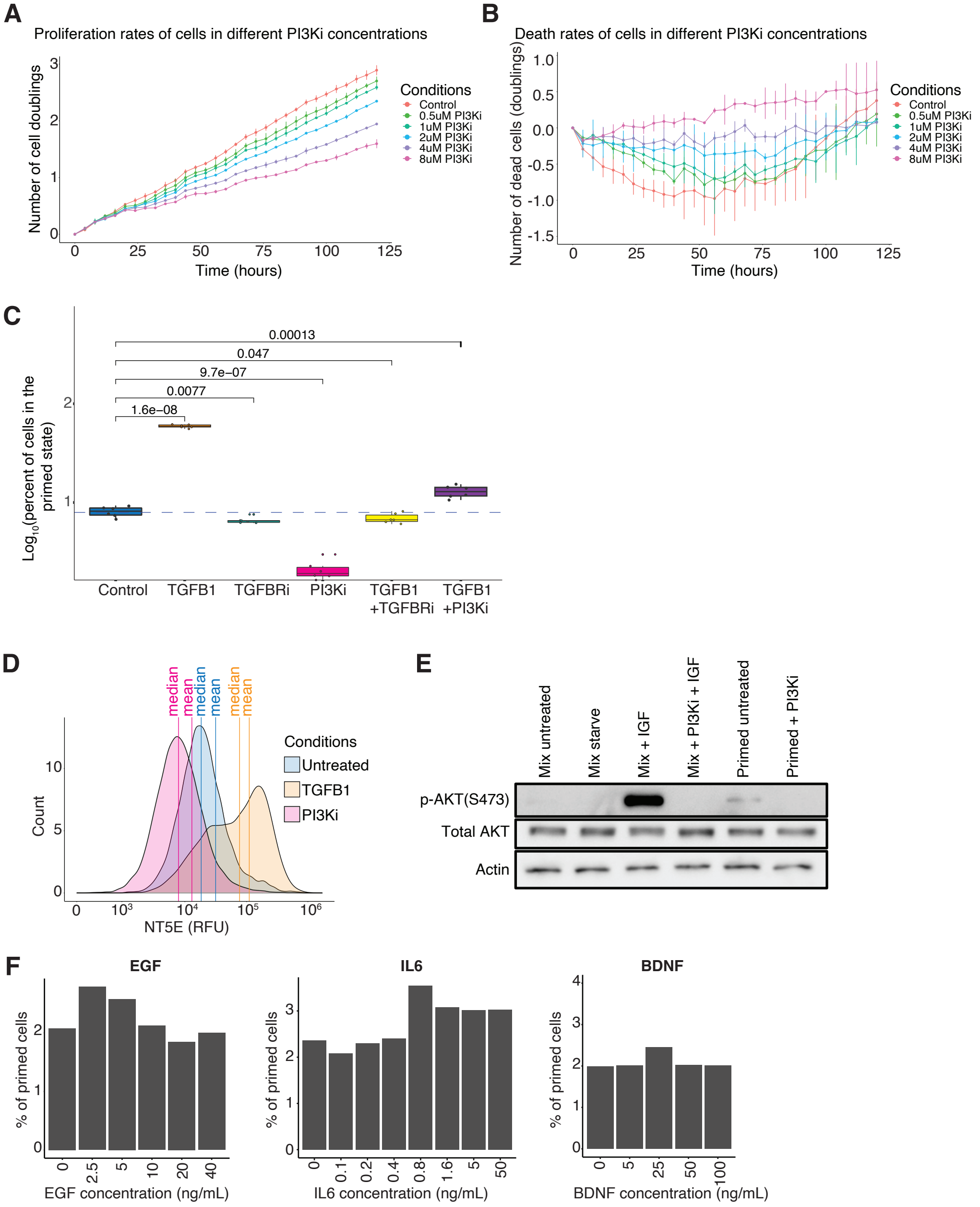

Supplementary Figure 4: TGFB1 and PI3Ki can modulate the percentage of primed cells. A. Line graph plotting the number of cell doublings over 5 days with different doses of PI3Ki. This data indicates that 2μM PI3Ki does not have a large impact on cell proliferation rate. Each point is the average of 6 technical replicates and error bars represent the mean absolute deviation. B. Line graph plotting the doubling of cell death for each concentration of PI3Ki (doubling is used to normalize for the amount of death at the initial time point). This data indicates that 2μM PI3Ki does not have a big impact on cell viability or proliferation rate. Each point is the average of 6 technical replicates and error bars represent the mean absolute deviation. C. Box plot of the log10 percent of WM989 cells in the primed state after 5 days in each respective treatment. This plot is the same data shown in Fig. 4C, but the threshold for calling a primed cell was set at 8% instead of 2%. The 2% threshold in Fig. 4C is the experimentally validated threshold for the enrichment of primed cells with NT5E, and 8% is the percent of primed cells found in the scRNA-seq data. Both thresholds show an increase in the percent of primed cells with TGFB1 and a decrease with PI3Ki. All conditions have 3 biological replicates each with 6 technical replicates. P values were calculated using a Wilcoxon test. The dotted line represents the mean log10 percent of primed (set at 8%) cells in the untreated condition. In this plot the center line is the median, the box is the IQR, and the whiskers indicate 1.5 times the upper and lower IQR. All data points are shown. D. Example histograms of NT5E expression as determined by flow cytometry in different treatments (this is the same data shown in Fig. 4B). The mean and median of each distribution are labeled. Both the mean and median of these populations shift, indicating that response to treatment is not due to large shifts in a few cells but that the whole population changes state due to treatment. E. Western blot showing the phosphorylation of AKT of different WM989 populations in different treatments. Mix population consists of sorting all live cells, and primed cells are sorted top 2% NT5E expressing cells. This experiment was performed with two biological replicates with consistent results. F. Bar graphs showing the effect of treating with different growth factors and cytokines (EGF, IL6, and BDNF) on the percent of primed cells in the population based on NT5E measurements by flow cytometry. These experiments show that the increase in primed cells caused by TGFB1 does not occur with these treatments.

Supplementary Figure 5

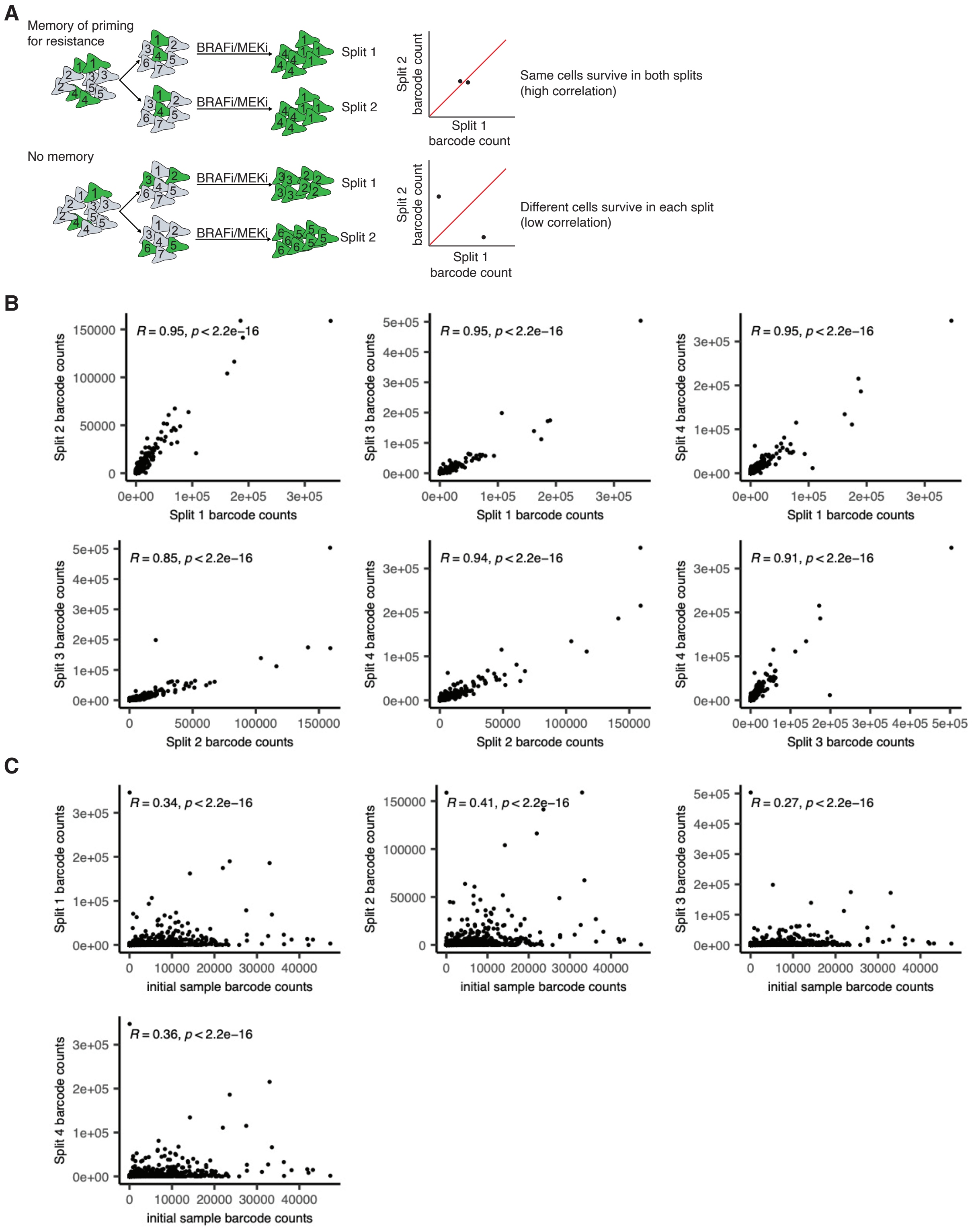

Supplementary Figure 5: The primed state can maintain memory through 8 cell divisions. A. Schematic showing the experimental design we used to assess whether there is memory underlying resistance to BRAFi/MEKi over 7-8 doublings (the number of cells shown in the diagram is less than 7-8 doublings for simplicity). In this assay, we transduced cells with the lentiviral barcode library (the barcodes are represented by the number in the cell) and then allowed the cells to divide through 7-8 doublings, so there are multiple cells with the same barcode. We then split the cells across multiple plates such that each split has cells with each barcode. We then treated each split with BRAFi/MEKi for 4 weeks and recovered the barcodes from gDNA. From the barcode sequences, we can determine whether the same cells survived in each split. The top schematic shows a scenario in which resistance has memory and the bottom schematic shows a scenario in which resistance does not have memory. In the top schematic, the same cells survive in each split leading to the same barcodes being recovered. This outcome leads to highly correlated barcode frequencies in both splits. When memory is not maintained, as shown in the bottom schematic, cells with different barcodes survive in each split, and therefore barcode abundance in both splits do not correlate. B. Results of a memory testing experiment described in A where barcoded cells were split into 4 plates after 7-8 doublings and treated with BRAFi/MEKi for 4 weeks. Scatter plots show a pairwise comparison of barcode frequencies in each split. The high correlation in these plots indicated memory was conserved through these divisions. Plots labeled with Pearson correlation and P values calculated as two-tailed t-test. C. Data from the same experiment in B, but a sampling of barcodes was taken before cells were treated with BRAFi/MEKi. These scatter plots show the abundance of barcodes before treatment with BRAFi/MEKi on the x-axes, and the barcode abundances in each split on the y-axes. Plots labeled with Pearson correlation and P values calculated as two-tailed t-test. This data shows that barcode frequencies before adding BRAFi/MEKi does not correlate with barcode frequencies after treatment. Thus, BRAFi/MEKi selects for a specific cell state, rather than merely selecting for the most abundant barcodes.

Supplementary Figure 6

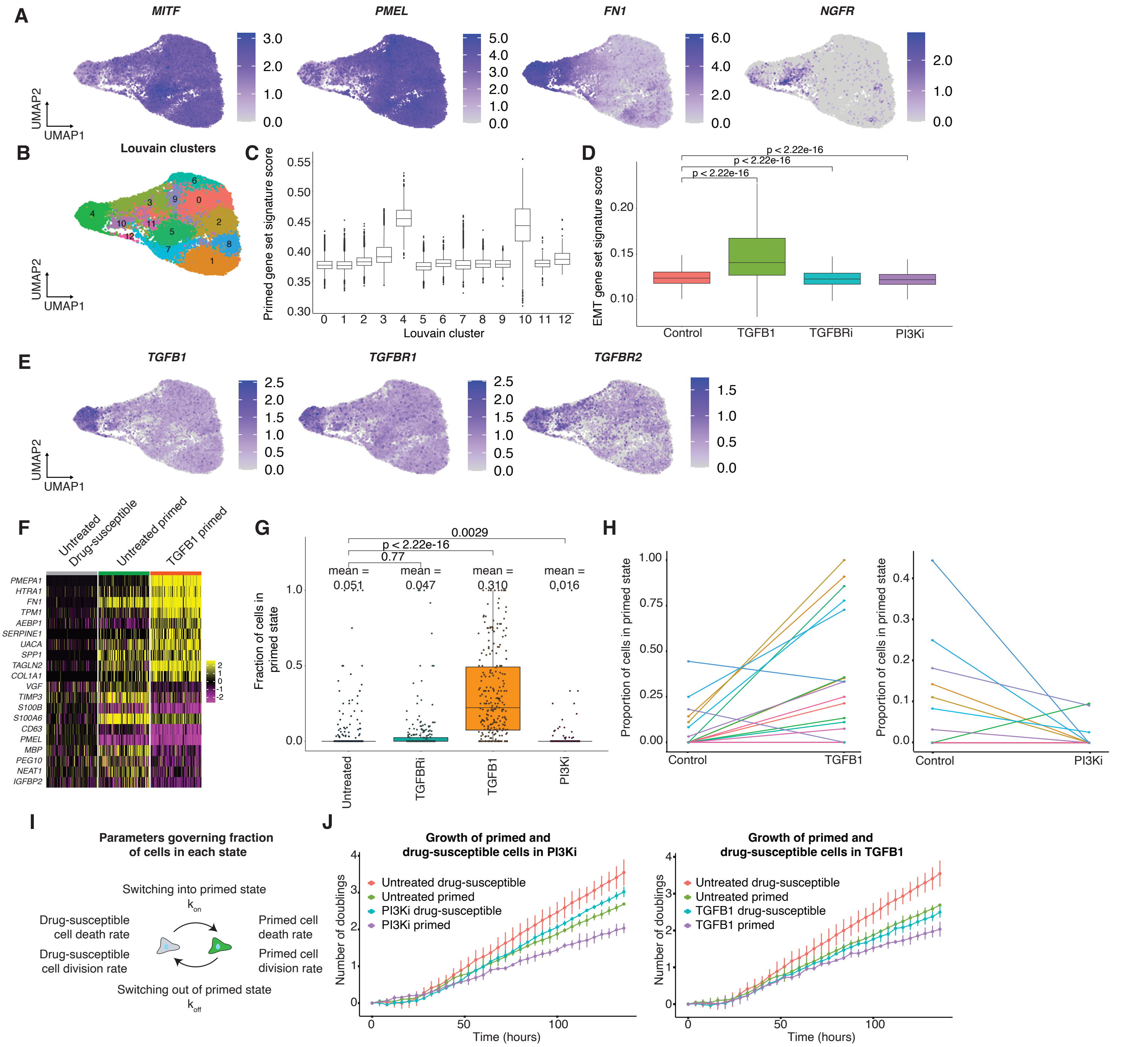

Supplementary Figure 6: Effects of TGFB1 and PI3Ki on gene expression and proliferation rates. A. UMAP plots showing normalized gene expression of *MITF*, *PMEL*, *FN1*, and *NGFR* for each cell. The gene expression patterns are very similar to those of scRNA-seq of WM989 cells in Supp. Fig. 1D. *MITF* and *PMEL* are generally anticorrelated with *FN1* and *NGFR*. B. UMAP plot showing Louvain clusters of all cells across all treatments from Fig. 4A. C. Box plot of the primed cell gene set signature score for each Louvain cluster. Clusters 4 and 10 have distinctly higher primed state signature scores and are thus classified as the primed state. Each data point represents an individual cell in the data set. D. Box plot of the EMT state signature score for cells in each treatment condition. This shows that TGFB1 treatment induced EMT genes. Each data point represents an individual cell in the data set, P values calculated using a two-sided Wilcoxon rank sum test. E. UMAP plots showing normalized gene expression of *TGFB1*, *TGFBR1*, and *TGFBR2*. These TGF-β signaling-associated genes are again high in the primed state. Notably, TGFB1-treated primed cells have high levels of expression of *TGFB1*, *TGFBR1*, and *TGFBR2*. F. Heatmap of gene expression for example genes in a representative sample of cells from the untreated drug-susceptible, untreated primed, and TGFB1-induced primed cells. The heatmap depicts log10 normalized and scaled gene expression. We find for most primed cell genes that the TGFB1-induced primed cells have higher expression than the untreated primed cells. Notably, there are a few genes upregulated in untreated primed cells that are not upregulated in the TGFB1-treated primed cells including *VGF* and *S100A6*. G. Box plot of the fraction of lineages in the primed state in each condition. Each data point is a lineage from the data set. Only cells with at least 2 cells per lineage per condition were included in this plot. TGFB1 and PI3Ki produce a statistically significant increase and decrease in the fraction of primed cells, respectively. P values calculated using a two-sided Wilcoxon rank sum test. H. Paired dot plots showing that the proportion of cells from a lineage in the primed state changes depending on the pretreatment received. The same colored dots connected by a line are data points from the same lineage. These plots show 20 randomly selected lineages. I. Schematic of the variables tested in our model to identify different ways changes in the number of primed cells can occur. The parameters tested include: the drug-susceptible state death and proliferation rates, primed state death and proliferation rates, the transition rate from the drug-susceptible state to the primed state (k on), and the transition rate from the primed state to the drug-susceptible state (k off) J. To measure the effects of TGFB1 and PI3Ki on the proliferation of each cell population, we sorted drug-susceptible and primed cells into separate wells and then treated them with TGFB1 or the PI3Ki (untreated control included). To track proliferation, we performed time-lapse microscopy over 5 days and counted nuclei every 4 hours. Plots show the doubling of nuclei in each condition. Error bars are the mean absolute deviation across 6 replicate wells. We find that both drug-susceptible cells and primed cells slow their proliferation in the presence of TGFB1 or the PI3Ki, but continue to divide. For the box plots in C, D, and G, the center line is the median, the box is the IQR, and the whiskers indicate 1.5 times the upper and lower IQR. All data points are shown.

Supplementary Figure 7

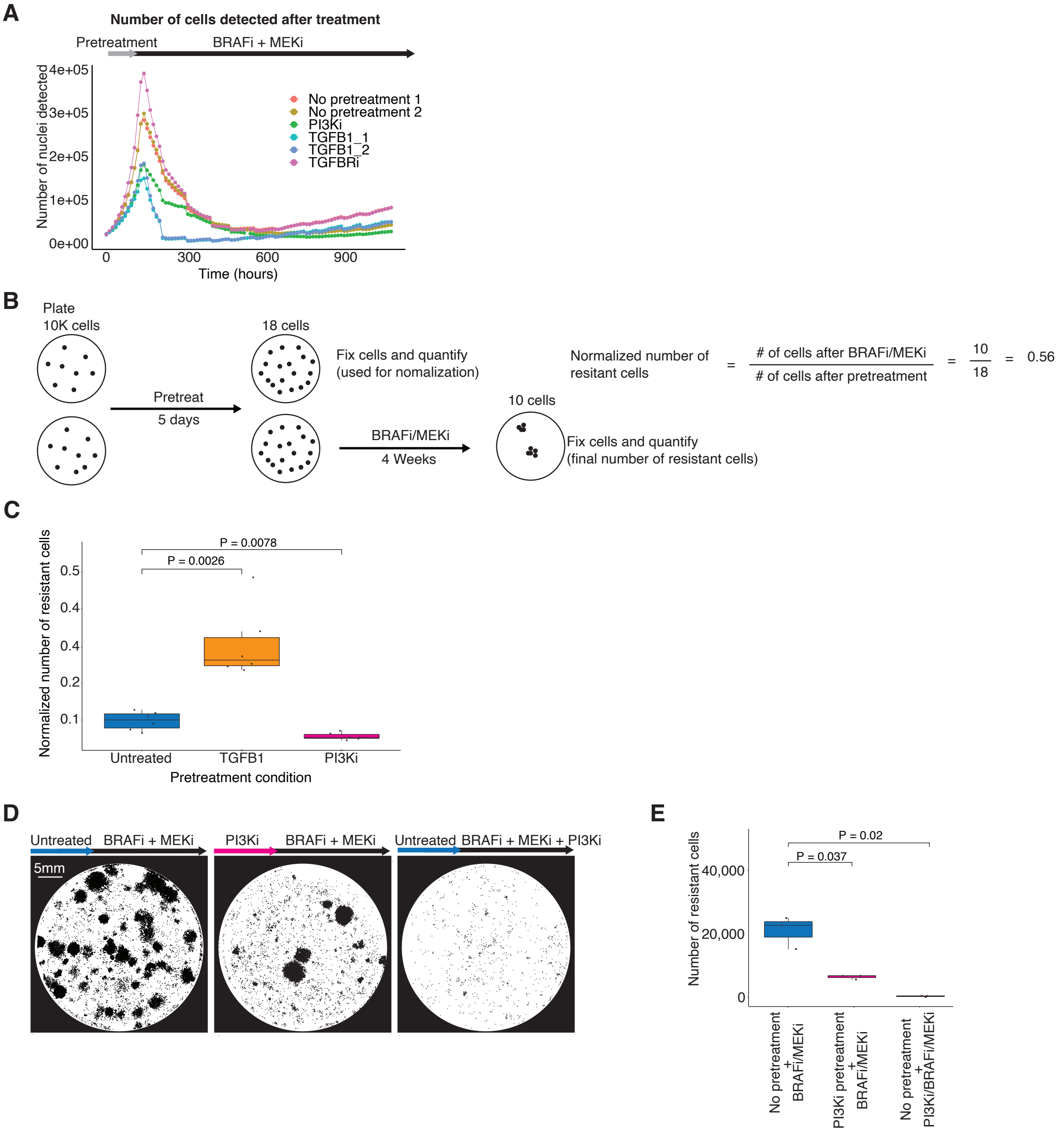

Supplementary Figure 7: Modulating the number of primed cells affects the resistant fate. A. Quantification of live-cell imaging data. Cells were treated with their respective pretreatment for 5 days and then treated with BRAFi/MEKi for 40 days. Plots show the number of cells detected at each time point. Untreated and TGFB1 conditions have 2 replicates each, the TGFBRi and the PI3Ki conditions have one replicate each. B. Diagram showing the experimental design used to normalize for the effects of pretreatments on proliferation. For each replicate, we treated 9 wells with the pretreatment condition (only one shown in the diagram). After pretreatment 3 wells were fixed to get the number of cells after pretreatment, and the other 6 were treated with BRAFi/MEKi for 4 weeks. C. Box plot showing the normalized number of resistant cells for the specified pretreatment condition. P values were calculated using a two-sided t-test, n=3 biological replicates. D. Example images of fixed cells stained with DAPI for each condition after 5 days of pretreatment and 5 weeks of selection with the specified treatment. The scale bar in the first image represents 5mm and applies to all images in the panel, n=3 biological replicates with 6 technical replicates in each. E. Box plot quantifying the number of drug-resistant cells from scans like those shown in panel B across n=3 biological replicates, each with 6 technical replicates. P values were calculated using a two-sided t-test. For the box plots in C and E, the center line is the median, the box is the IQR, and the whiskers indicate 1.5 times the upper and lower IQR. All data points are shown.

Supplementary Figure 8:

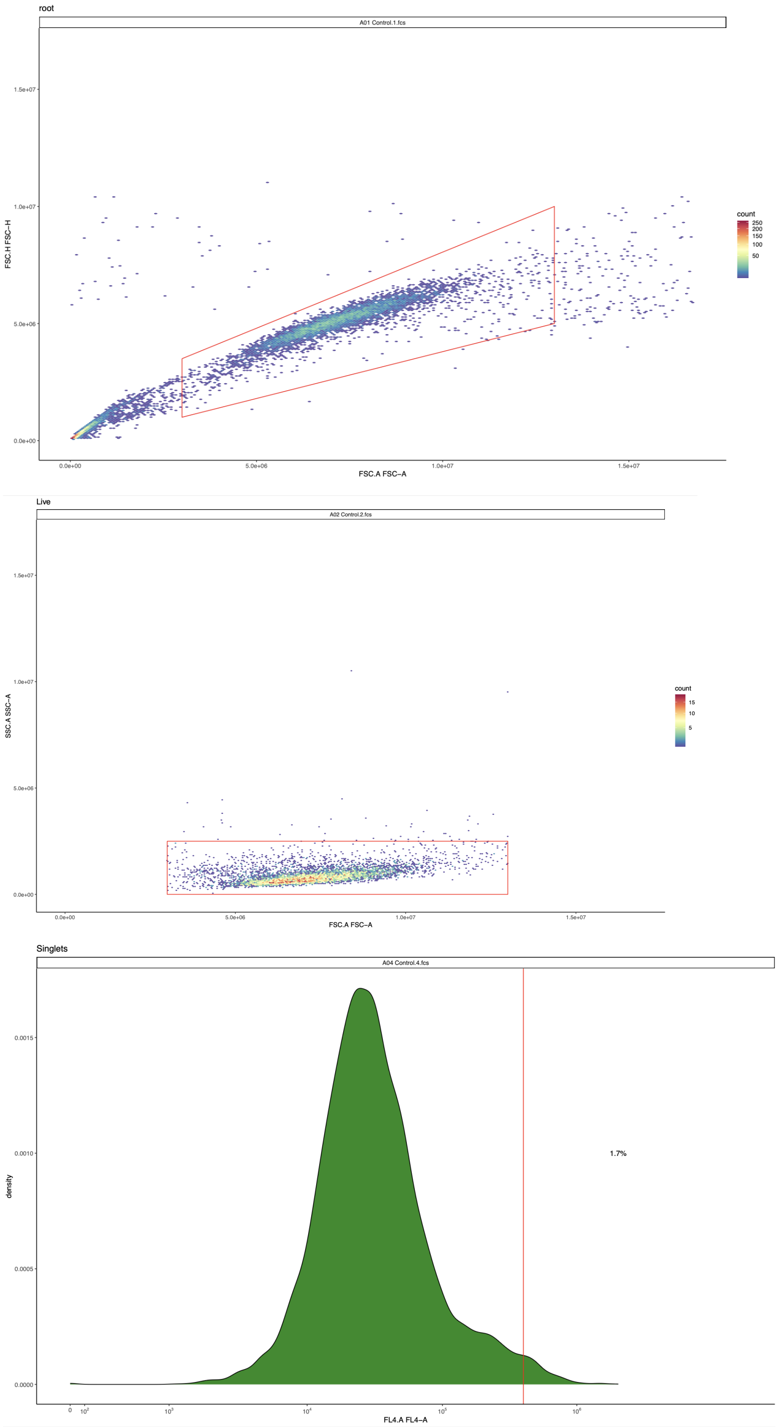

Supplementary Figure 8: Example of FACS sequential gating strategy. Stepwise example gating for sorting based on expression of NT5E, NGFR, and/or EGFR. First gating to identify cells, next subsetting for singlets, then selecting the top percentage of the target. Different thresholds for NT5E, NGFR, and EGFR are specified for each experiment and described in the methods.

Full blot for Supplementary Figure 4E:

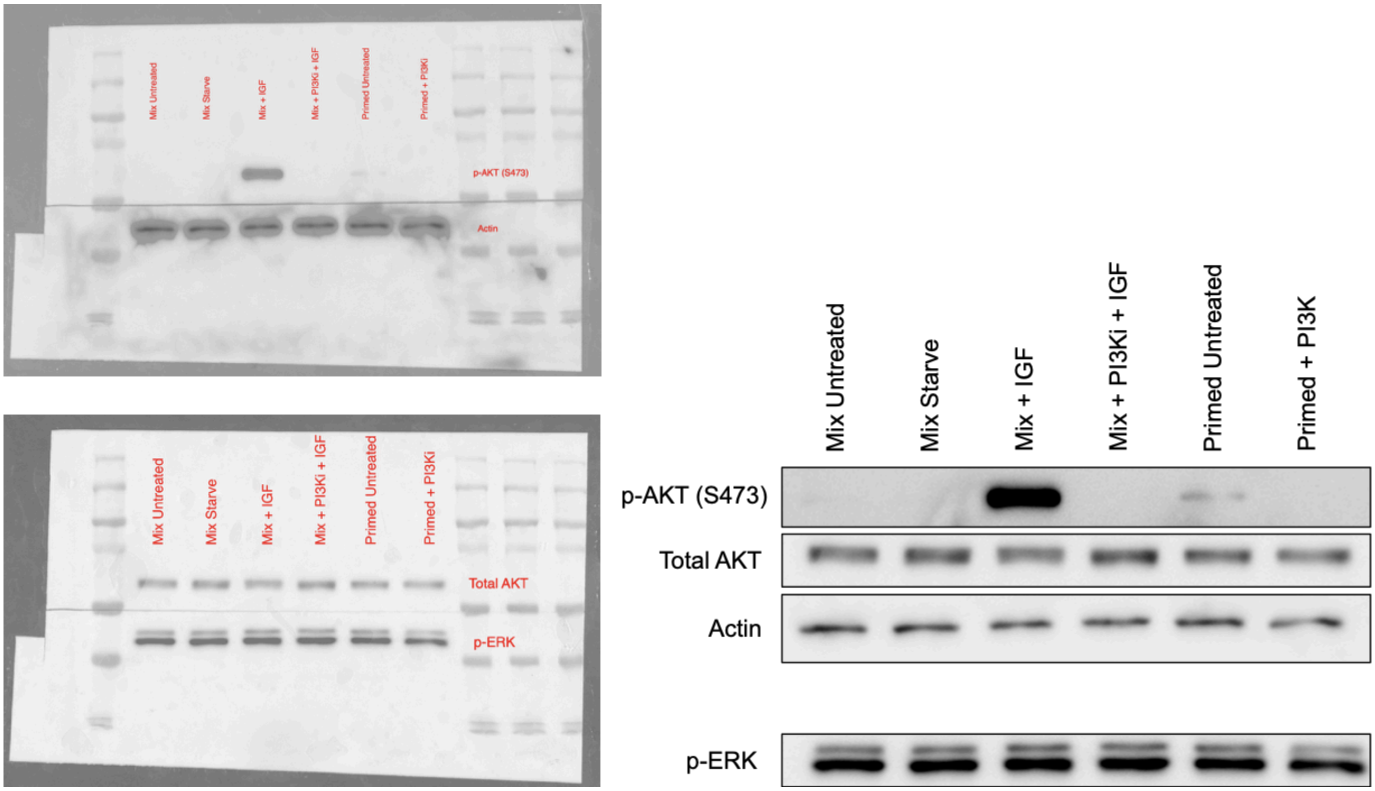

Replicate data:

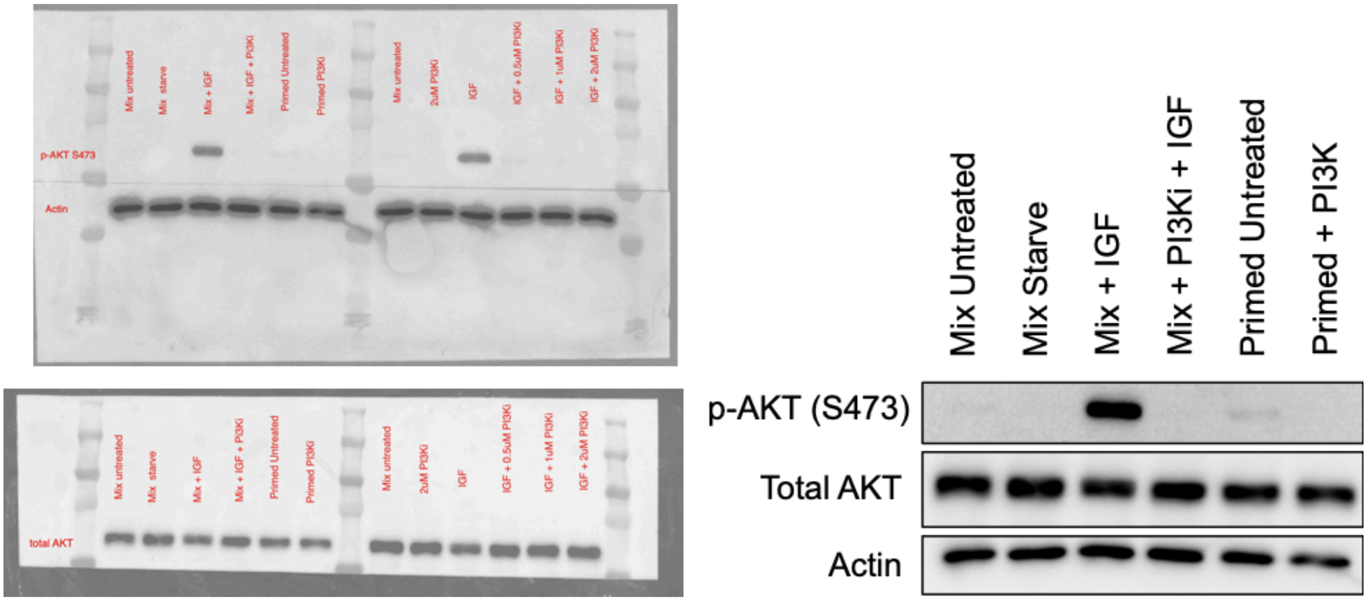

Supplementary Figure 9: Full blots from Supplementary Figure 4E and replicates.
The sizes for the ladder on the whole figure are 250 > 150 > 100 > 75 > 50 > 37 > 25. We used Bio-Rad Precision Plus Dual Color Standards #1610374EDU.

Supplementary Table 1: Primers used to amplify lineage barcodes.

Primers used to amplify lineage barcodes from 10X cDNA<br>sequence in red is the index sequence	GH_i5.1.1	AATGATACGGCGACCACCGAGATCTACACCTAGCGCTACACTCTTTCCCTACACGACGCTCTTCCGATCT
	GH_i5.2.1	AATGATACGGCGACCACCGAGATCTACACTCGATATCACACTCTTTCCCTACACGACGCTCTTCCGATCT
	GH_i7.3.1	CAAGCAGAAGACGGCATACGAGATGTCTCTACGTGACTGGAGTTCAGACGTGTGCTCTTCCGATCTAGGACGAGCTGTACAAGTAGG
	GH_i7.3.2	CAAGCAGAAGACGGCATACGAGATCAGATAGTGTGACTGGAGTTCAGACGTGTGCTCTTCCGATCTCTGGACGAGCTGTACAAGTAGG
	GH_i7.3.3	CAAGCAGAAGACGGCATACGAGATTCTACGCAGTGACTGGAGTTCAGACGTGTGCTCTTCCGATCTTGCGGACGAGCTGTACAAGTAGG
	GH_i7.3.4	CAAGCAGAAGACGGCATACGAGATCGACTCTGGTGACTGGAGTTCAGACGTGTGCTCTTCCGATCTGCTCGGACGAGCTGTACAAGTAGG
Primers used to amplify lineage barcodes from gDNA<br>sequence in red is the index sequence<br>blue sequence is a "Stagger sequece" so that	GHi5.2.1	AATGATACGGCGACCACCGAGATCTACACTAGATCGCACACTCTTTCCCTACACGACGCTCTTCCGATCTNHNNNNTCGACTAAACGCGCTACTTG
	GHi5.2.2	AATGATACGGCGACCACCGAGATCTACACCTCTCTATACACTCTTTCCCTACACGACGCTCTTCCGATCTNHNNNNCGTCGACTAAACGCGCTACTTG
	GHi5.2.3	AATGATACGGCGACCACCGAGATCTACACTATCCTCTACACTCTTTCCCTACACGACGCTCTTCCGATCTNHNNNNGACTCGACTAAACGCGCTACTTG
	GHi5.2.4	AATGATACGGCGACCACCGAGATCTACACAGAGTAGAACACTCTTTCCCTACACGACGCTCTTCCGATCTNHNNNNATAGTCTCGACTAAACGCGCTACTTG
	GHi5.2.5	AATGATACGGCGACCACCGAGATCTACACGTAAGCAGACACTCTTTCCCTACACGACGCTCTTCCGATCTNHNNNNTACGTCGACTAAACGCGCTACTTG
	GHi5.2.6	AATGATACGGCGACCACCGAGATCTACACACTGCGTAACACTCTTTCCCTACACGACGCTCTTCCGATCTNHNNNNCCTAATCGACTAAACGCGCTACTTG
	GHi5.2.7	AATGATACGGCGACCACCGAGATCTACACAAGGAGTAACACTCTTTCCCTACACGACGCTCTTCCGATCTNHNNNNTCGACTAAACGCGCTACTTG
	GHi5.2.8	AATGATACGGCGACCACCGAGATCTACACCTAAGCCTACACTCTTTCCCTACACGACGCTCTTCCGATCTNHNNNNGCTCGACTAAACGCGCTACTTG
	GHi5.2.9	AATGATACGGCGACCACCGAGATCTACACCGTCTAATACACTCTTTCCCTACACGACGCTCTTCCGATCTNHNNNNCTGTCGACTAAACGCGCTACTTG
	GHi5.2.10	AATGATACGGCGACCACCGAGATCTACACTCTCTCCGACACTCTTTCCCTACACGACGCTCTTCCGATCTNHNNNNTATCAGTCGACTAAACGCGCTACTTG
	GHi5.2.11	AATGATACGGCGACCACCGAGATCTACACTCGACTAGACACTCTTTCCCTACACGACGCTCTTCCGATCTNHNNNNATGCTCGACTAAACGCGCTACTTG
	GHi5.2.12	AATGATACGGCGACCACCGAGATCTACACTTCTAGCTACACTCTTTCCCTACACGACGCTCTTCCGATCTNHNNNNGGATATCGACTAAACGCGCTACTTG
	GHi7.2.1	CAAGCAGAAGACGGCATACGAGATTAAGGCGAGTGACTGGAGTTCAGACGTGTGCTCTTCCGATCTGTCCTGCTGGAGTTCGTGAC
	GHi7.2.2	CAAGCAGAAGACGGCATACGAGATCGTACTAGGTGACTGGAGTTCAGACGTGTGCTCTTCCGATCTAGGTCCTGCTGGAGTTCGTGAC
	GHi7.2.3	CAAGCAGAAGACGGCATACGAGATAGGCAGAAGTGACTGGAGTTCAGACGTGTGCTCTTCCGATCTTCAAGTCCTGCTGGAGTTCGTGAC
	GHi7.2.4	CAAGCAGAAGACGGCATACGAGATTCCTGAGCGTGACTGGAGTTCAGACGTGTGCTCTTCCGATCTCACTTAAGTCCTGCTGGAGTTCGTGAC
	GHi7.2.5	CAAGCAGAAGACGGCATACGAGATCGACTCCTGTGACTGGAGTTCAGACGTGTGCTCTTCCGATCTTGTCCTGCTGGAGTTCGTGAC
	GHi7.2.6	CAAGCAGAAGACGGCATACGAGATTAGGCATGGTGACTGGAGTTCAGACGTGTGCTCTTCCGATCTCTTGAGTCCTGCTGGAGTTCGTGAC
	GHi7.2.7	CAAGCAGAAGACGGCATACGAGATATCGCTACGTGACTGGAGTTCAGACGTGTGCTCTTCCGATCTGTAGTCCTGCTGGAGTTCGTGAC
	GHi7.2.8	CAAGCAGAAGACGGCATACGAGATCAGAGAGTGTGACTGGAGTTCAGACGTGTGCTCTTCCGATCTGATACTGTCCTGCTGGAGTTCGTGAC

Supplementary Information

Michael Saint-Antoine and Abhyudai Singh

1 Introduction

As discussed previously in this paper, we are interested in studying the dynamics of melanoma cells that switch back and forth between two transient transcriptional states: a "drug-susceptible" state in which they die off when exposed to the drug and a "primed" state in which they survive exposure to the drug. In this section, we discuss the results of an experiment in which populations of these cells were exposed to different treatments, to study their effects on the fraction of cells in the primed state. We will then use stochastic simulations to model different possible mechanisms of these treatments, to see which model scenarios yield results that most closely match the real data.

The experimental procedure was as follows. A population of cells was grown out for approximately 7-8 generations. The population was then split into two groups. One group was exposed to a treatment (either TGFB1, TGFBRi, or PI3Ki), while the other group was left as an untreated control. Both the treated and untreated groups were then grown out for approximately 2 more generations, and then finally sequenced to reveal the transcriptomic state of each cell. Throughout the experiment, the lineages of the cells were tracked. This allows us to compare the fraction of primed cells in the treated group and the untreated group for each lineage. When analyzing these results, we apply a threshold so that only lineages with at least 5 cells total are considered, and this same threshold was applied to the simulation results of our stochastic model.

Supplementary Table 2: Fraction of Cells in Primed State – Treated vs. Untreated Statistics

	**TGFB1**		**TGFBRi**		**PI3Ki**	
	**Untreated**	**Treated**	**Untreated**	**Treated**	**Untreated**	**Treated**
**Mean**	0.0342	0.2900	0.0328	0.0353	0.0328	0.0079
**Std. Deviation**	0.1130	0.2615	0.1076	0.1081	0.1092	0.0451
**CV**	3.3032	0.9019	3.2794	3.0611	3.3330	5.7077
**Fano Factor**	0.3732	0.2359	0.3529	0.3308	0.3639	0.2576

The results of this experiment are shown in Supplementary Table 2. Here we consider the fractions of primed cells for each lineage in the treated and untreated groups, and report the mean, standard deviation, coefficient of variation, and Fano factor for these fractions across lineages. Note that the untreated groups in all three experimental cases show very similar statistics – a sign that the control groups are working as expected. Comparing the treated and untreated mean primed fractions in each case allows us to see the effects of the treatments. In the case of TGFB1, the treatment increased the fraction of cells in the primed state. In the case of TGFBRi, the fraction of primed cells in the treated and untreated groups were very similar. In the case of PI3Ki, the treatment decreased the fraction of cells in the primed state.

Based on this experiment, we can clearly see the effects of each treatment. However, the mechanisms that are giving rise to these effects are still unknown. Take, for example, the case of the TGFB1 treatment. We can see that the treatment is increasing the fraction of cells in the primed state, but how exactly is this happening? Is the treatment increasing the rate at which cells switch into the primed state? Or is it decreasing the rate at which cells switch out of the primed state? Or could it be having some effect on

the proliferation rates? To study this, we use a stochastic model of the experimental cell population to run computer simulations of different possible scenarios to see which simulated scenarios most closely match the real data from the experiment. We apply this analysis to the TGFB1 and PI3Ki cases. Please note that this should be understood as a hypothesis-generating analysis, not a hypothesis-testing analysis. That is, the conclusions we draw from the model are not definitive confirmations of the true underlying mechanisms of each treatment, but rather clues that could help us design future experiments. The experimental data and Python code used for this analysis are available upon request from the authors.

2 Model Formulation

Let us consider a population of cells that proliferate and switch back and forth between the drug-susceptible state and the primed state. We use the integer-valued stochastic processes $s(t)$ and $p(t)$ to represent the number of cells in the drug-susceptible state and the number of cells in the primed state, respectively. Drug-susceptible cells proliferate with rate r_1 and primed cells proliferate with rate r_2. Drug-susceptible cells switch to the primed state with rate k_{on} (we also call this "switching on") and primed cells switch back to the drug-susceptible state with rate k_{off} ("switching off"). We refer to the average fraction of primed cells in the untreated state as f_{exp}, and this number is slightly different for the different experimental cases.

Supplementary Table 3 shows the stochastic formulation of this model. Each row is an event that can occur in the cell population, requiring an update of $s(t)$ and $p(t)$, which keep track of the number of cells in each state. Each event occurs with the appropriate rate and propensity. We can use this setup to simulate the model using the stochastic simulation algorithm [1].

Supplementary Table 3: Stochastic Model

Event	Var. Updates	Rate	Propensity
Drug-susceptible proliferation	$s \to s+1$	r_1	$r_1 \cdot s$
Primed proliferation	$p \to p+1$	r_2	$r_2 \cdot p$
Drug-susceptible to primed	$s \to s-1$<br>$p \to p+1$	k_{on}	$k_{on} \cdot s$
Primed to drug-susceptible	$s \to s+1$<br>$p \to p-1$	k_{off}	$k_{off} \cdot p$

Before fitting this model to data, we make some simplifying assumptions in order to reduce the number of independent parameters we need to fit. First, we set the drug-susceptible state proliferation rate $r_1 = 1$, so that time in our model is normalized to the average generation time of cells in the drug-susceptible state. So, our time unit is average drug-susceptible state generations, rather than hours, days, etc.

Next, we make a simplifying assumption in order to write k_{on} in terms of k_{off}. Consider a scenario in which a large population of cells exists in a steady state, with no proliferation, and an average fraction of $\hat{f}$ in the primed state at any given time. Under these theoretical conditions, $\hat{f}$ can be written in terms of the state-switching rates as:

$$\hat{f} = \frac{k_{on}}{k_{on} + k_{off}} \tag{1}$$

The intuition here is that if we know one of the switch-rates and the average primed fraction in the population, we can figure out what the other switch rate must be in order to maintain that equilibrium. We make the simplifying assumption that the average primed fraction across lineages in our experimental data is approximately equal to this underlying theoretical steady state primed fraction: $f_{exp} \approx \hat{f}$. Making this assumption allows us to write the switch-on rate in terms of the switch-off rate and the average primed fraction:

$$k_{on} = \frac{k_{off}}{\frac{1}{f_{exp}} - 1} \tag{2}$$

A consequence of defining k_{on} this way is that different experimental data sets can have different fractions in the primed state, meaning different values for k_{on}. That is why the k_{on} values for the TGFB1 and PI3Ki treatment scenarios are slightly different in the following sections.
After normalizing the model time units to average drug-susceptible generation time and making our simplifying assumption to write equation (2), we are left with only two independent parameters to fit to the data: r_2 and k_{off}.

3 Estimating Untreated Parameters

The next step in our analysis is to estimate parameters for the untreated cells. To do this, we use data from another experiment in which cells were grown out for approximately 4 generations, and then sequenced to reveal their transcriptional state. Again, the lineages of the cells were tracked, so we can look at the numbers of drug-susceptible and primed cells from the same original lineage.

We use a modified minimum least-squares method to fit our untreated parameters to this data set. First, we consider a deterministic version of the stochastic model proposed in the previous section:

$$\frac{ds}{dt} = r_1 s - k_{on} s + k_{off} p \tag{3}$$

$$\frac{dp}{dt} = r_2 p + k_{on} s - k_{off} p \tag{4}$$

We then make the same simplifying assumptions described in the previous section, setting $r_1 = 1$ and $k_{on} = \frac{k_{off}}{\frac{1}{f_{exp}} - 1}$. In the case of this experimental data set, the average fraction of primed cells across lineages was 0.125, so $f_{exp} = 0.125$. Plugging in this value gives us $k_{on} = \frac{k_{off}}{7}$. So, the system of equations is:

$$\frac{ds}{dt} = s - \frac{k_{off}}{7} s + k_{off} p \tag{5}$$

$$\frac{dp}{dt} = r_2 p + \frac{k_{off}}{7} s - k_{off} p \tag{6}$$

In our model, we assume that a cell population is grown out from a single original cell, which could be either drug-susceptible or primed. So, this system of ODEs has two possible initial conditions: $(s(0) = 1, p(0) = 0)$ or $(s(0) = 0, p(0) = 1)$. We use the function notation $s_0(r_2, k_{off}), p_0(r_2, k_{off})$ to denote the solutions to the ODEs at $t = 4$, with the initial condition of one drug-susceptible cell and zero primed cells. We use the function notation $s_1(r_2, k_{off}), p_1(r_2, k_{off})$ to denote the solutions to the ODEs at $t = 4$, with the initial condition of zero drug-susceptible cells and one primed cell.

We refer to the experimental drug-susceptible and primed cell counts for each lineage as $\hat{s}$ and $\hat{p}$, respectively. We define a loss function as follows. For each experimental data point, we calculate the squared errors for the drug-susceptible and primed counts, and add them together, assuming the initial condition $(s(0) = 1, p(0) = 0)$. We then do the same calculation, assuming the initial condition $(s(0) = 0, p(0) = 1)$. After calculating both of these measures, we take the minimum of the two and add that to our total loss. We do this for each experimental data point. The mathematical formulation of this loss function is:

$$l(r_2, k_{off}) = \sum_{i=1}^{N} \min\left\{(s_0(r_2, k_{off}) - \hat{s}_i)^2 + (p_0(r_2, k_{off}) - \hat{p}_i)^2, (s_1(r_2, k_{off}) - \hat{s}_i)^2 + (p_1(r_2, k_{off}) - \hat{p}_i)^2\right\} \tag{7}$$

Then, we simply apply a numerical optimization in Python to find the values of r_2 and $koff$ that minimize the loss function.

$$\min_{r_2, k_{off}} \quad l(r_2, k_{off}) \tag{8}$$

Supplementary Table 4 shows estimates for these parameter values that we have obtained through this method. We use a bootstrapping technique, running the analysis 40 times on random samples of the original data set, in order to generate a distribution of results. We report the 2.5 percentile value, median, and 97.5 percentile value of the results, so that the range between the lower bound and upper bound includes 95% of observed parameter fit values. The data and Python code used for the fitting are available upon request. Please note that these parameter values should be taken as rough estimates, not as exact, certain values.

Supplementary Table 4: Estimated Parameter Values

**Event description**	**Parameter**	**Fit Value**		
		**95% Lower Bound**	**Median**	**95% Upper Bound**
Primed cell proliferation	r_2	0.3714	0.4786	0.5605
Primed to drug-susceptible switch	k_{off}	0.1247	0.1666	0.2158

4 Experiment Simulation

As discussed previously, we have data from an experiment in which cells were grown out for a period of time and then divided into two groups, with some given a treatment (TGFB1, TGFBRi, or PI3Ki), while others were left untreated. Since lineages were tracked throughout this experiment, we can compare the fraction of cells in the primed state for both treated and untreated cells within the same lineage. For example, Supplementary Figure 10 is a plot of the fraction of treated cells in the primed state against the fraction of untreated cells in the primed state, for the TGFB1 experiment. Each point represents these fractions for a single lineage.

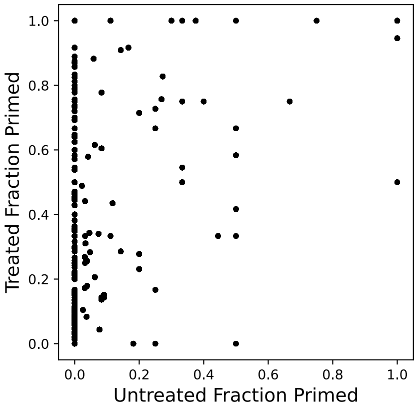

Supplementary Figure 10: Fraction of primed treated cells vs. fraction of primed untreated cells per lineage, for the TGFB1 treatment case.

Our goal is to use the stochastic model described previously to generate simulated datasets like this for different possible mechanisms of the treatments, and then see how closely the simulated results for different possible scenarios fit the real data.

We simulate the experiment as follows. We begin with a single cell, which is randomly chosen to be in

the primed state with probability f_{exp} or to be in the drug-susceptible state with probability $1 - f_{exp}$. The cell is grown out into a colony from $t = 0$ until $t = 7$ (approximately 7 drug-susceptible state generations in our model). During this time, the cells proliferate and switch back and forth between the drug-susceptible and primed states. For this part of the simulation, we use the fitted untreated parameters listed in Supplementary Table 4. At the end of this portion of the simulation, we are left with a simulated population of cells, which may be drug-susceptible, primed, or a mix. At this point, we divide the simulated cell population in half, making the simplifying assumption that they are divided exactly in half, which both divisions having exactly the same number of primed cells and exactly the same number of drug-susceptible cells. When an odd number interferes with this, we round the quotient to make this division feasible.

From this point, we use the two groups of simulated cells to simulate the second part of the experiment, in which one group is exposed to the treatment while the other is left untreated. In terms of our simulation, we continue to use the same parameters for the untreated group. For the treated group, we continue the simulation, but change one of the parameters to model a possible mechanism of the treatment. The process of selecting these new, treated parameter values is described in the next section.

5 Estimating Treated Parameters

As explained in the previous section, we model the effect of the treatment by changing one of the parameters, while keeping every other parameter the same as in the untreated case. For different scenarios, we change different parameters. Our ultimate goal is to compare the log-likelihood scores of different scenarios to see which ones can better explain the data. However, before we can compare across different scenarios, we must first select the treated parameter values for each case. We use a truncated, rough approximation of the maximum log-likelihood method to choose these parameter values.

Let us quickly review the concept of log-likelihood. Consider a set of experimental observations $X_1, X_2, ..., X_N$. We can attempt to model the system that yielded these observations, using a set of input parameters θ that yields a probability distribution $f_X(X, \theta)$. For a given parameter set θ, we can calculate the log-likelihood of observing the data points:

$$\sum_{i=1}^{N} log(f_X(X_i, \theta)) \tag{9}$$

Here log is the natural logarithm. Then, to fit our model to the data, our task is to search through the space of possible parameter values to find the values of θ that maximize this measure of log-likelihood.

We use this approach to estimate the new treated parameter value for each treatment scenario. Since we do not have an exact analytical solution for the joint probability distribution of the prime fractions of treated and untreated cells in our model, we estimate this distribution empirically, by running many simulations and binning the results. This involves running the simulated experiment many times for each new parameter value being tested, so the process is quite computationally expensive and has a considerable runtime. Due to this computational constraint, we estimate the empirical probability distributions for each parameter set using only 1000 runs of the experiment simulation, a smaller than ideal number. This also leads to another computational problem – when empirically estimating the distributions, it is possible to have a bin with 0 observations in the model and at least 1 observation in the experimental data. This causes a calculation error, since we cannot take the natural logarithm of 0. So, when binning the results of simulations, we assume that each bin starts at 1, not 0, and count observations from there. To ensure that this computational shortcut is not skewing the results, we will drop it in the following section when we validate the log-likelihood scores for each scenario by using 1000000 runs of the model to generate the distributions.

Using this log-likelihood parameter estimation method, we test 400 potential new parameter values for each scenario, chosen from a uniform distribution, from the previous untreated parameter value to some upper or lower bound. We save the value that yields the highest log-likelihood score, and use that as our

new treated parameter value for that scenario. The new parameter values for each scenario are shown in the following two tables.

Supplementary Table 5: TGFB1 - Treated Parameter Estimation

**Scenario**	**Changed Parameter**	**Untreated Value**	**Treated Value**
Drug-Susceptible Proliferation Decrease	r_1	1	0.0166
Primed Proliferation Increase	r_2	0.4786	2.9059
Switch-On Rate Increase	k_{on}	$\frac{k_{off}}{\frac{1}{f_{exp}}-1} = 0.0059$	0.0855
Dependent Switch-On Rate	k_{on}	$\frac{k_{off}}{\frac{1}{f_{exp}}-1} = 0.0059$	$0.0013 \cdot p$
Switch-Off Rate Decrease	k_{off}	0.1666	0.0651

Supplementary Table 6: PI3Ki - Treated Parameter Estimation

**Scenario**	**Changed Parameter**	**Untreated Value**	**Treated Value**
Drug-Susceptible Proliferation Increase	r_1	1	2.2954
Primed Proliferation Decrease	r_2	0.4786	0.0473
Switch-On Rate Decrease	k_{on}	$\frac{k_{off}}{\frac{1}{f_{exp}}-1} = 0.0056$	0.0012
Switch-Off Rate Increase	k_{off}	0.1666	3.3485

6 TGFB1 Treatment

We have observed that when the cell populations are treated with TGFB1, that yields an increased fraction of cells in the primed state compared to an untreated control. However, the underlying mechanism behind this is not known. We consider five possible scenarios that could yield an increase in the fraction of cells in the primed state:

1. A decrease in the drug-susceptible proliferation rate r_1
2. An increase in the primed proliferation rate r_2
3. An increase in the switch-on rate k_{on}
4. A decrease in the switch-off rate k_{off}
5. A scenario in which the switch-on rate k_{on} depends on the number of primed cells p

For each of these possible scenarios, we ran a simulation of the experiment 1000000 times, and binned the results in order to generate an empirical probability distribution. We used this probability distribution of simulated results to calculate a log-likelihood score for each scenario. Note that in terms of log-likelihood, a higher number means a better fit, and a more negative number means a worse fit. The results are shown in Supplementary Table 7, listed in order of best-fit to worst-fit.

Supplementary Figure 11 shows a visual representation of these results. For each plot of treated prime cell fractions to untreated prime cell fractions, the back dots show real data points from the experimental dataset. The blue shading shows the distribution of simulation results for each scenario. The shading is not exactly propor-tional to the distribution frequency, and has been adjusted to saturate at a level below the maximum to

Supplementary Table 7: TGFB1 - Simulation Results

Scenario	Changed Parameter	Log-Likelihood Score
Primed Proliferation Increase	r_2	-50563.78
Dependent Switch-On Rate	k_{on}	-54531.503
Drug-Susceptible Proliferation Decrease	r_1	-84862.688
Switch-On Rate Increase	k_{on}	-88256.291
Switch-Off Rate Decrease	k_{off}	-112885.818

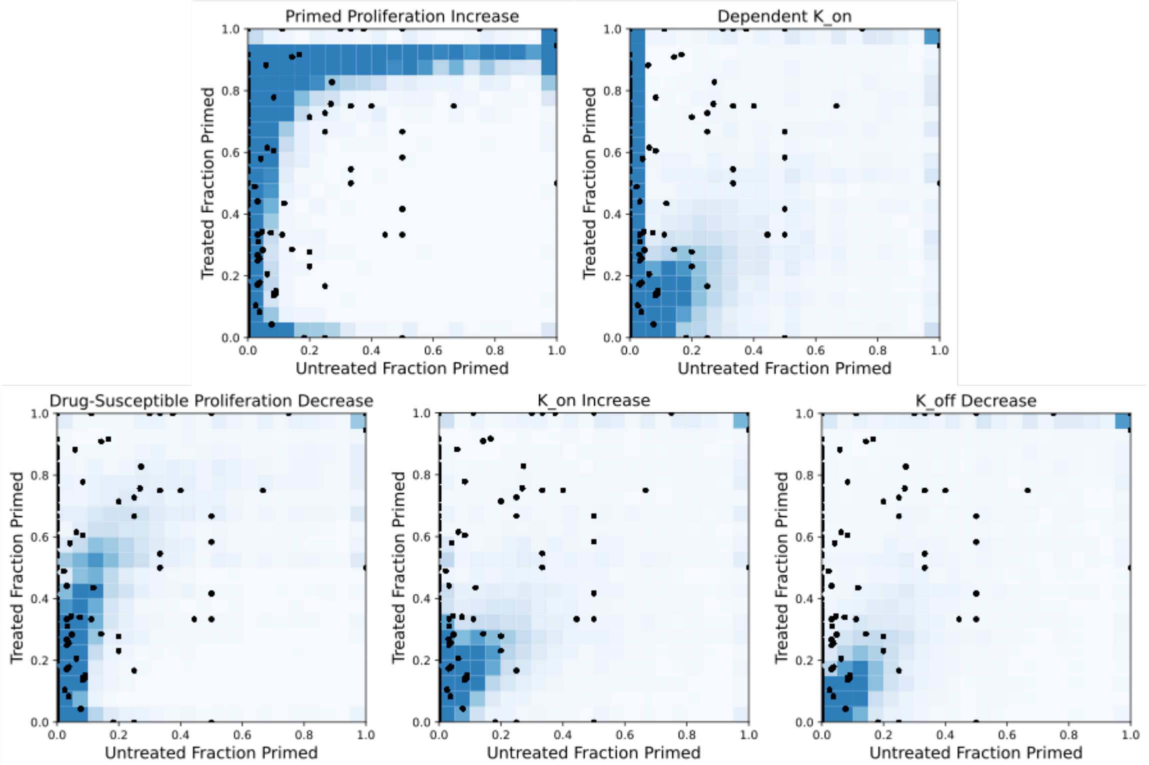

Supplementary Figure 11: Fraction of primed treated cells vs. fraction of primed untreated cells per lineage, for the TGFB1 treatment case. Real data points, shown in black dots, are plotted over the distribution of simulation results, shown with blue shading.

highlight simulation outliers (in order to better to compare to the real data point outliers, which are visible).

Please note that many of the real experimental data points in these plots lie along the vertical axis (this may be difficult to see). We believe that the difference in the log-likelihood scores between scenarios is largely the result of their relative ability or inability to account for these data points. The Primed Proliferation Increase and Dependent Switch-On Rate scenarios are able to account for these data points along the vertical axis (as can be seen by their dark blue shaded bars along the vertical axis), while the Drug-Susceptible Proliferation Decrease, Switch-On Rate Increase, and Switch-Off Rate Decrease scenarios do not account for them as well.

7 PI3Ki Treatment

In the PI3Ki treatment data, we observed a decrease in the fraction of cells in the primed state in the treated sample compared to the untreated control. The underlying mechanism behind this is not known. In this section, we consider four possible scenarios:

1. An increase in the drug-susceptible proliferation rate r_1
2. A decrease in the primed proliferation rate r_2
3. A decrease in the switch-on rate k_{on}
4. An increase in the switch-off rate k_{off}

We applied the same analytical workflow here as in the TGFB1 analysis, running a simulation of the experiment 1000000 times and binning the results in order to generate an empirical probability distribution. Again, we used this simulated probability distribution to calculate a log-likelihood score for each scenario. These results are shown in Supplementary Table 8, in order of best-fit to worst-fit.

Supplementary Table 8: PI3Ki - Simulation Results

Scenario	Changed Parameter	Log-Likelihood Score
Drug-Susceptible Proliferation Increase	r_1	-10544.337
Switch-Off Rate Increase	k_{off}	-10991.598
Primed Proliferation Decrease	r_2	-11017.362
Switch-On Rate Decrease	k_{on}	-11774.535

Supplementary Figure 12 shows a visual representation of these results, plotting the treated prime cell fractions against the untreated prime cell fractions. The black dots show real data points from the experimental dataset, and the blue shading shows the distribution of simulation results for each scenario. As with Supplementary Figure 11, the shading in Supplementary Figure 12 is not exactly proportional to the distribution frequency, and has been adjusted to saturate at a level below the maximum to highlight simulation outliers.

Although it is slightly difficult to see visually, we note that many of the real experimental data points lie along the horizontal axis, and the relative goodness or badness of fit for the scenarios seems to be determined by their ability or inability to account for these data points.

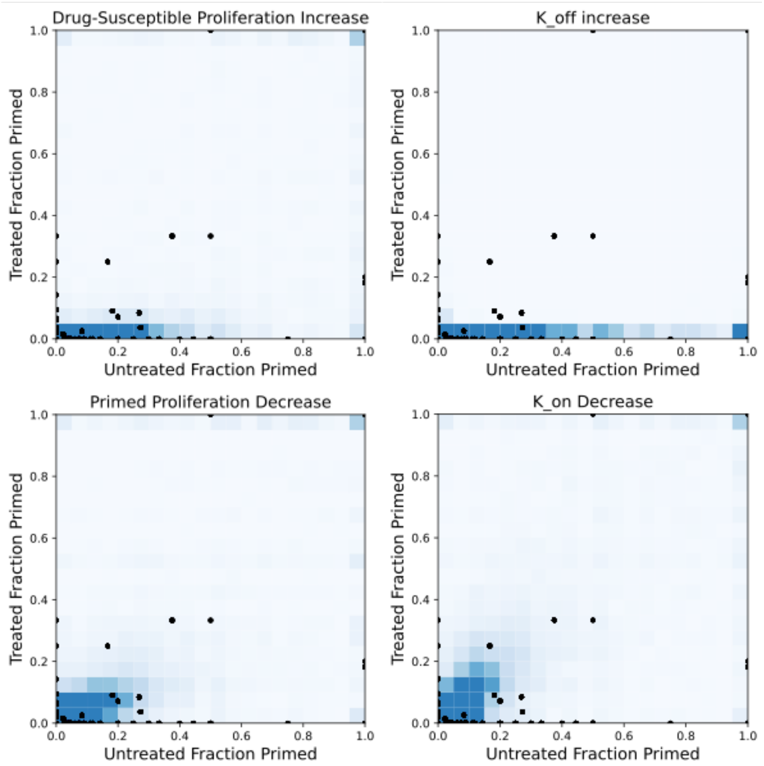

Supplementary Figure 12: Fraction of primed treated cells vs. fraction of primed untreated cells per lineage, for the PI3Ki treatment case. Real data, shown in black dots, are plotted over the distribution of simulation results, shown with blue shading.

Supplementary References

1. Rambow, F. *et al.* Toward Minimal Residual Disease-Directed Therapy in Melanoma. *Cell* **174**, 843–855.e19 (2018).
2. Karras, P. *et al.* A cellular hierarchy in melanoma uncouples growth and metastasis. *Nature* vol. 610 190–198 Preprint at https://doi.org/10.1038/s41586-022-05242-7 (2022).
3. Tirosh, I. *et al.* Dissecting the multicellular ecosystem of metastatic melanoma by single-cell RNA-seq. *Science* **352**, 189–196 (2016).
4. Jerby-Arnon, L. *et al.* A Cancer Cell Program Promotes T Cell Exclusion and Resistance to Checkpoint Blockade. *Cell* **175**, 984–997.e24 (2018).
5. Belote, R. L. *et al.* Human melanocyte development and melanoma dedifferentiation at single-cell resolution. *Nat. Cell Biol.* **23**, 1035–1047 (2021).
6. Shaffer, S. M. *et al.* Memory Sequencing Reveals Heritable Single-Cell Gene Expression Programs Associated with Distinct Cellular Behaviors. *Cell* (2020) doi:10.1016/j.cell.2020.07.003.
