# Supplementary material for: Disrupting cellular memory to overcome drug resistance
Source: Nat Commun. 2023 Nov 6;14:7130. doi: 10.1038/s41467-023-41811-8 (PMC10628298; doi:10.1038/s41467-023-41811-8)
Supplement: Supplementary file 3 — Reporting Summary [file 41467_2023_41811_MOESM3_ESM.pdf]

nature portfolio

Corresponding author(s): Sydney Shaffer

Last updated by author(s): 8/2/2023

Reporting Summary

Nature Portfolio wishes to improve the reproducibility of the work that we publish. This form provides structure for consistency and transparency in reporting. For further information on Nature Portfolio policies, see our Editorial Policies and the Editorial Policy Checklist.

Statistics

For all statistical analyses, confirm that the following items are present in the figure legend, table legend, main text, or Methods section.

n/a	Confirmed	
☐	☒	The exact sample size (*n*) for each experimental group/condition, given as a discrete number and unit of measurement
☐	☒	A statement on whether measurements were taken from distinct samples or whether the same sample was measured repeatedly
☐	☒	The statistical test(s) used AND whether they are one- or two-sided<br>*Only common tests should be described solely by name; describe more complex techniques in the Methods section.*
☒	☐	A description of all covariates tested
☐	☒	A description of any assumptions or corrections, such as tests of normality and adjustment for multiple comparisons
☐	☒	A full description of the statistical parameters including central tendency (e.g. means) or other basic estimates (e.g. regression coefficient) AND variation (e.g. standard deviation) or associated estimates of uncertainty (e.g. confidence intervals)
☐	☒	For null hypothesis testing, the test statistic (e.g. *F*, *t*, *r*) with confidence intervals, effect sizes, degrees of freedom and *P* value noted<br>*Give P values as exact values whenever suitable.*
☒	☐	For Bayesian analysis, information on the choice of priors and Markov chain Monte Carlo settings
☒	☐	For hierarchical and complex designs, identification of the appropriate level for tests and full reporting of outcomes
☒	☐	Estimates of effect sizes (e.g. Cohen's *d*, Pearson's *r*), indicating how they were calculated

Our web collection on statistics for biologists contains articles on many of the points above.

Software and code

Policy information about availability of computer code

Data collection	For fluorescence activated cell sorting we used Summit (version 62) software. For flow cytometry we used the Accuri C6 software (version 264). For timelapse of cells we used the Incucyte software (version 2021C).
Data analysis	For analysis of single-cell RNA sequencing we used cellranger (version 3.1) to generate count matrices from the raw data. For further analysis of the single-cell RNA sequencing we used Seurat (version 3) and UCell (version 2). For flow cytometry data analysis we used flowCore (version 2.8). For nuclear segmentation we used DeepCell (version 0.12.2). For nucleus counting we used custom sofware called NucID (can be found on GitHub). For ATAC alignment we used bowtie2 (version 2.3.4.1), filtered out low quality read alignments using samtools (version 1.1), removed duplicated reads with picard (version 1.96), and called peaks with MACS2 (version 2.1.1.20160309). For further analysis of the ATAC data we used consensusSeekeR (version 1.24). For counting drug resistant colonies we used custom software called ColonySelector (can be found on GitHub). For lineage barcode analysis we used custom software called BarcodeAnalysis (can be found on GitHub).

For manuscripts utilizing custom algorithms or software that are central to the research but not yet described in published literature, software must be made available to editors and reviewers. We strongly encourage code deposition in a community repository (e.g. GitHub). See the Nature Portfolio guidelines for submitting code & software for further information.

Data

Policy information about availability of data

All manuscripts must include a data availability statement. This statement should provide the following information, where applicable:

- Accession codes, unique identifiers, or web links for publicly available datasets
- A description of any restrictions on data availability
- For clinical datasets or third party data, please ensure that the statement adheres to our policy

The scRNA-seq data generated in this study have been deposited in Gene Expression Omnibus under accession code GSE237228. The publicly available data used in this study is available in the GEO database under accession code GSE115978, GSE77940 (Jerby-Arnon et al. 2018). The remaining data are available within the Article, Supplementary Information or Source Data file.
All data and code used for this paper can be found here:
https://drive.google.com/drive/folders/1-C78090Z43w5kGb1ZW8pXgysjha35jlU?usp=sharing

Research involving human participants, their data, or biological material

Policy information about studies with human participants or human data. See also policy information about sex, gender (identity/presentation), and sexual orientation and race, ethnicity and racism.

Reporting on sex and gender	N/A
Reporting on race, ethnicity, or other socially relevant groupings	N/A
Population characteristics	N/A
Recruitment	N/A
Ethics oversight	N/A

Note that full information on the approval of the study protocol must also be provided in the manuscript.

Field-specific reporting

Please select the one below that is the best fit for your research. If you are not sure, read the appropriate sections before making your selection.

☒ Life sciences ☐ Behavioural & social sciences ☐ Ecological, evolutionary & environmental sciences

For a reference copy of the document with all sections, see nature.com/documents/nr-reporting-summary-flat.pdf

Life sciences study design

All studies must disclose on these points even when the disclosure is negative.

Sample size	The sample size for single-cell RNA sequencing was determined based on cell loading instructions from 10X genomics. All other sample sizes were selected to show the reproducibility of results, no sample-size calculations were performed.
Data exclusions	Data exclusion was used in the analysis to remove doublets and dead cells. These were determined by UMI counts and mitochondrial gene percentage respectively (see methods for more details). These exclusion criteria were preestablished.
Replication	We performed biological and technical replicates for all experiments. The number of replicates are specified for each experiment in the figure captions. See the methods section for more details about the number of replicates used for each experiment. All results were reproducible.
Randomization	Randomization is not relevant to this study because the same clonal cell lines were used in all experimental groups.
Blinding	Blinding was not necessary as there was no group allocation.

Reporting for specific materials, systems and methods

We require information from authors about some types of materials, experimental systems and methods used in many studies. Here, indicate whether each material, system or method listed is relevant to your study. If you are not sure if a list item applies to your research, read the appropriate section before selecting a response.

Materials & experimental systems

n/a	Involved in the study
☐	☒ Antibodies
☐	☒ Eukaryotic cell lines
☒	☐ Palaeontology and archaeology
☐	☒ Animals and other organisms
☒	☐ Clinical data
☒	☐ Dual use research of concern
☒	☐ Plants

Methods

n/a	Involved in the study
☒	☐ ChIP-seq
☐	☒ Flow cytometry
☒	☐ MRI-based neuroimaging

Antibodies

Antibodies used	NGFR primary antibody (Biolegend, 345108), EGFR primary antibody (Clone 225, fisher scientific, MABF120MI), NT5E primary antibody (Biolegend, 344005), Alexa Fluor 488 Donkey Anti-Mouse secondary antibody (Jackson Labs, 715-545-151), Phospho-AKT Ser473 (Cell Signaling Technology #4060), pan-AKT (Cell Signaling Technology #9272), b-actin (Santa Cruz Biotechnology #4778).
Validation	All antibodies used were validated by the manufacturer for the application and species they were used for in this paper.

Eukaryotic cell lines

Policy information about cell lines and Sex and Gender in Research

Cell line source(s)	WM989 (female) and WM983B (male) are patient derived cell lines that were provided to us by the Meenhard Herlyn Lab. HEK293FT cells were provided by the lab of Arjun Raj at UPenn.
Authentication	Cell lines were authenticated using STR profiling
Mycoplasma contamination	All cell lines were tested for Mycoplasma and tested negative
Commonly misidentified lines (See ICLAC register)	None

Animals and other research organisms

Policy information about studies involving animals; ARRIVE guidelines recommended for reporting animal research, and Sex and Gender in Research

Laboratory animals	NOD/SCID mice (Charles River Laboratories, Wilmington, MA)
Wild animals	N/A
Reporting on sex	Findings are not sex dependent
Field-collected samples	N/A
Ethics oversight	All animal experiments were approved by Wistar's Institutional Animal Care and Use Committee (IACUC) (IACUC #112503X_0) and were performed in an Association for the Assessment and Accreditation of Laboratory Animal Care (AAALAC) accredited facility.

Note that full information on the approval of the study protocol must also be provided in the manuscript.

Flow Cytometry

Plots

Confirm that:

☒ The axis labels state the marker and fluorochrome used (e.g. CD4-FITC).

☒ The axis scales are clearly visible. Include numbers along axes only for bottom left plot of group (a 'group' is an analysis of identical markers).

☒ All plots are contour plots with outliers or pseudocolor plots.

☒ A numerical value for number of cells or percentage (with statistics) is provided.

Methodology

Sample preparation	We dissociated cells from the plate using trypsin-EDTA into a single-cell suspension and washed once with 0.1% BSA. We then

Sample preparation	resuspended the cells in a 1:200 dilution of anti-NT5E antibody conjugated with APC and incubated them for 30min on ice. Next, we washed the cells once with 0.1% BSA, once with 1% BSA, and then resuspended them in 1% BSA for analysis by flow cytometry. We used an Accuri C6 for our flow cytometry and quantified 10,000 events per sample. To analyze the data we used the R package flowCore (Hahne et al. 2009).
Instrument	BD Accuri C6
Software	To analyze the data we used the R package flowCore (Hahne et al. 2009).
Cell population abundance	N/A
Gating strategy	In our analysis, we used forward and side scatter to identify cells, and used the FL4 channel (640nm excitation laser and 675/25 filter) to quantify cell surface levels of NT5E. To determine what percent of cells were primed, we set an intensity threshold where 2% of untreated cells would land above the threshold. We considered any cells above this threshold as primed.

☒ Tick this box to confirm that a figure exemplifying the gating strategy is provided in the Supplementary Information.
