# Supplementary material for: Disrupting cellular memory to overcome drug resistance
Source: Nat Commun. 2023 Nov 6;14:7130. doi: 10.1038/s41467-023-41811-8 (PMC10628298; doi:10.1038/s41467-023-41811-8)
Supplement: Supplementary file 4 — Description of Additional Supplementary Files [file 41467_2023_41811_MOESM4_ESM.pdf]

Description of additional supplementary files:

Supplementary Data 1: Intermediate state gene sets. Differential expression analysis comparing drug-susceptible cells in lineages that contain cells that switched state to cells in lineages containing only drug-susceptible cells. We found 575 genes differentially expressed between these types of lineages (based upon a differential gene expression analysis with a cutoff of 0.05 Bonferroni adjusted p-value and 0.25 log fold change cutoff). Differential expression analysis using Wilcoxon rank sum test, two-sided.

Supplementary Data 2: Differential expression comparing primed and drug susceptible state cells. Table of differentially expressed genes between primed and drug-susceptible state cells. Differential expression analysis using Wilcoxon rank sum test, two-sided.

Supplementary Data 3: Differential expression comparing cells that switched states in the presence of PI3Ki to cells that did not switch state. Table of differentially expressed genes. Differential expression analysis using Wilcoxon rank sum test, two-sided.

Supplementary Table 1: Primers used to amplify lineage barcodes. Table of primer sequences used for this study.

Supplementary Methods 1: Full description of mathematical modeling methods.
